# Supplementary material for: Targeting Gα13-integrin interaction ameliorates systemic inflammation
Source: Nat Commun. 2021 May 27;12:3185. doi: 10.1038/s41467-021-23409-0 (PMC8159967; doi:10.1038/s41467-021-23409-0)
Supplement: Supplementary file 1 — Supplementary Information [file 41467_2021_23409_MOESM1_ESM.pdf]

SUPPLEMENTARY INFORMATION

Targeting Gα$_{13}$-integrin interaction ameliorates systemic inflammation

Ni Cheng[1], Yaping Zhang[1], M. Keegan Delaney[1,2], Can Wang[1], Yanyan Bai[1], Randal A. Skidgel[2] and Xiaoping Du[1]*

[1] Department of Pharmacology, University of Illinois at Chicago College of Medicine, Chicago, IL 60612.

[2] DuPage Medical Technology, Inc. Chicago, IL 60612.

*To whom correspondence should be addressed: Xiaoping Du, Email: xdu@uic.edu.

This file includes: Supplementary Figures 1-9 and Supplementary table 1.

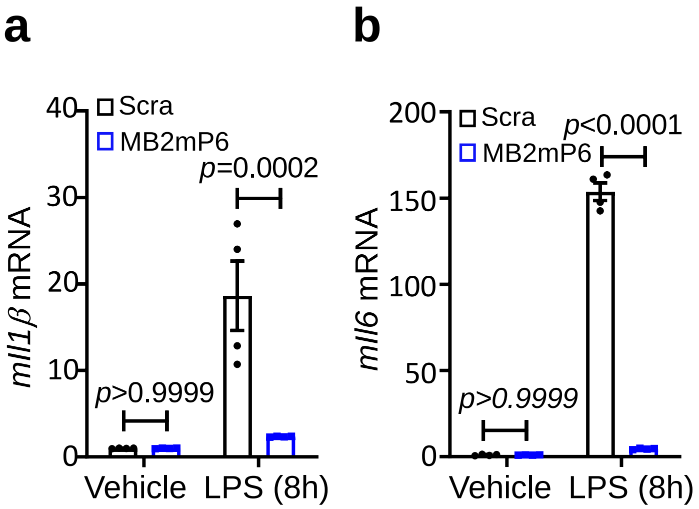

Supplementary Fig. 1. MB2mP6 inhibits expression of cytokines in mouse macrophages. Cytokine transcripts *il-1β* (**a**) and *il-6* (**b**) in mouse bone-marrow derived macrophages (BMDMs) were detected by qRT-PCR (all groups, n=4, independent cultures). Data are shown as mean ± SEM. Data were analyzed by two-way ANOVA with the post hoc Bonferroni's multiple comparisons test.

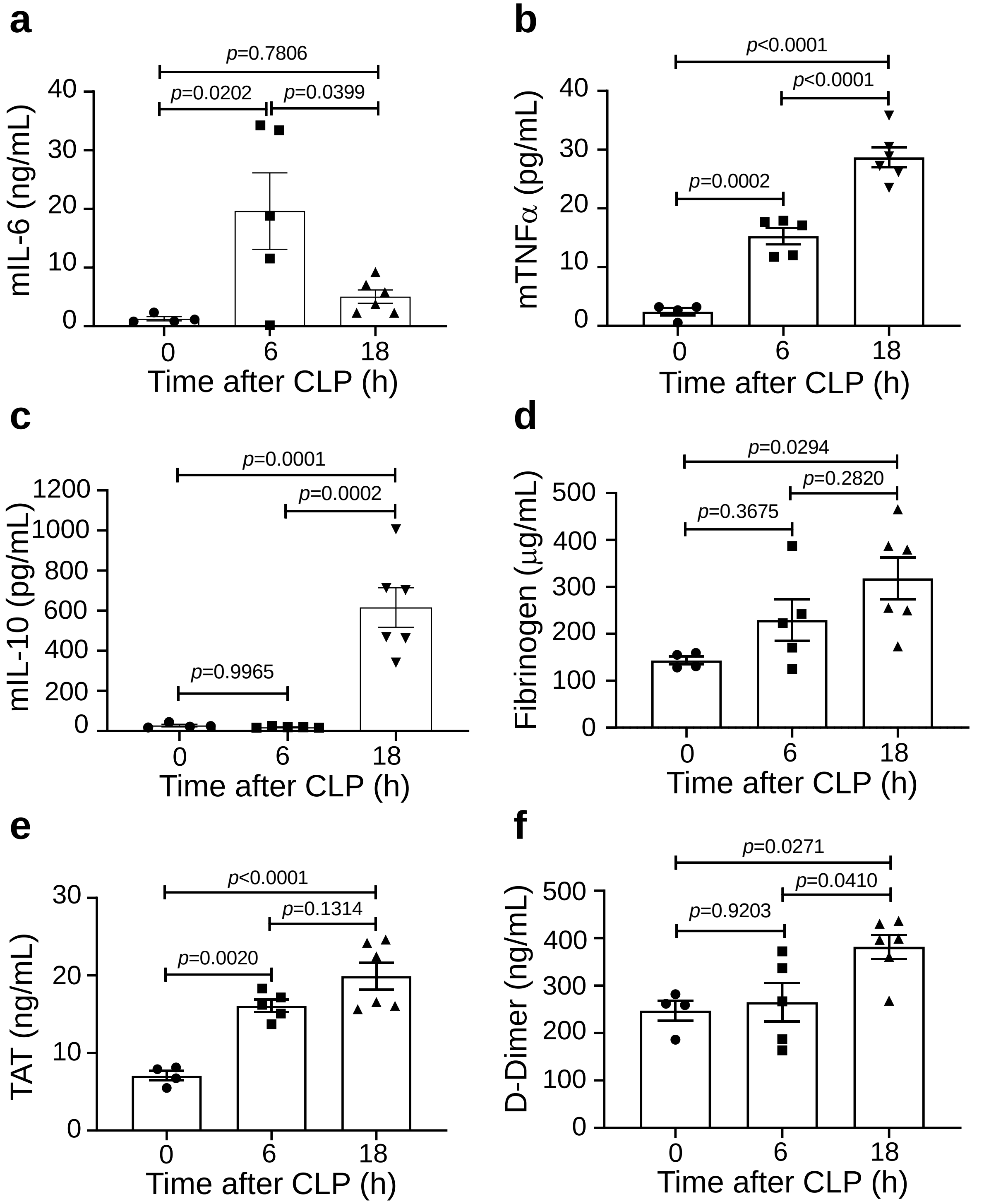

Supplementary Fig. 2. Detection of Cytokine and coagulation factor levels in septic mice 6 and 18 hours after CLP onset. Expression of selected cytokines IL-6 (**a**), TNFα (**b**) and IL-10 (**c**) at the protein level in mouse serum measured by ELISA 0, 6 and 18 hours after CLP. Coagulation factors fibrinogen (**d**), TAT (**e**) and fibrin degradation product D-Dimer (f) contents in mouse plasma examined by ELISA 0, 6 and 18 hours after CLP. All data are shown as mean ± SEM (0 h, n=4; 6 h, n=5; 18 h, n=6, independent animals). Data were analyzed by one-way ANOVA with the post hoc Tukey's multiple comparisons test.

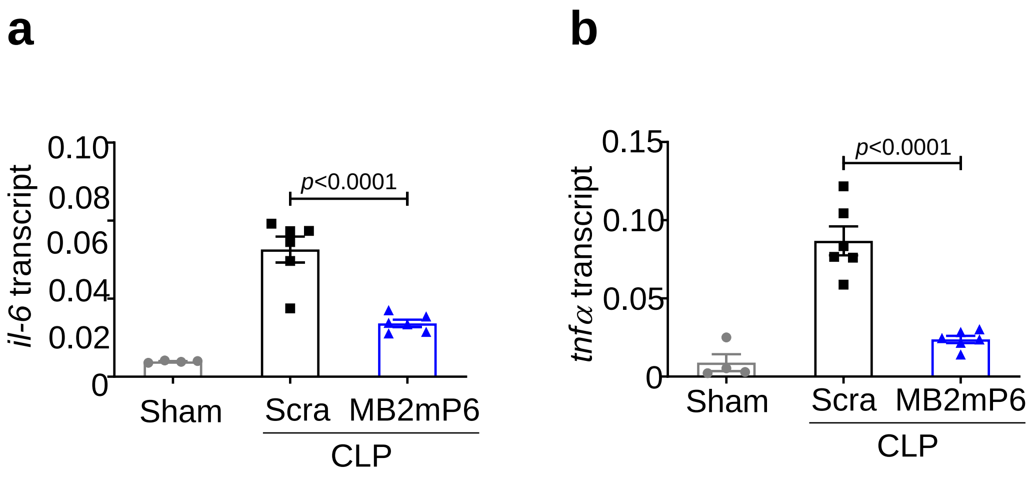

Supplementary Fig. 3. MB2mP6 inhibits expression of cytokine transcripts *il-6* (a) and *tnfα* (b) in septic mouse lungs 24 hours after CLP as detected by qRT-PCR. Data are shown as mean ± SEM (sham, n=4; Scra, n=6; MB2mP6, n=6, independent animals). Data were analyzed by one-way ANOVA with the post hoc Tukey's multiple comparisons test.

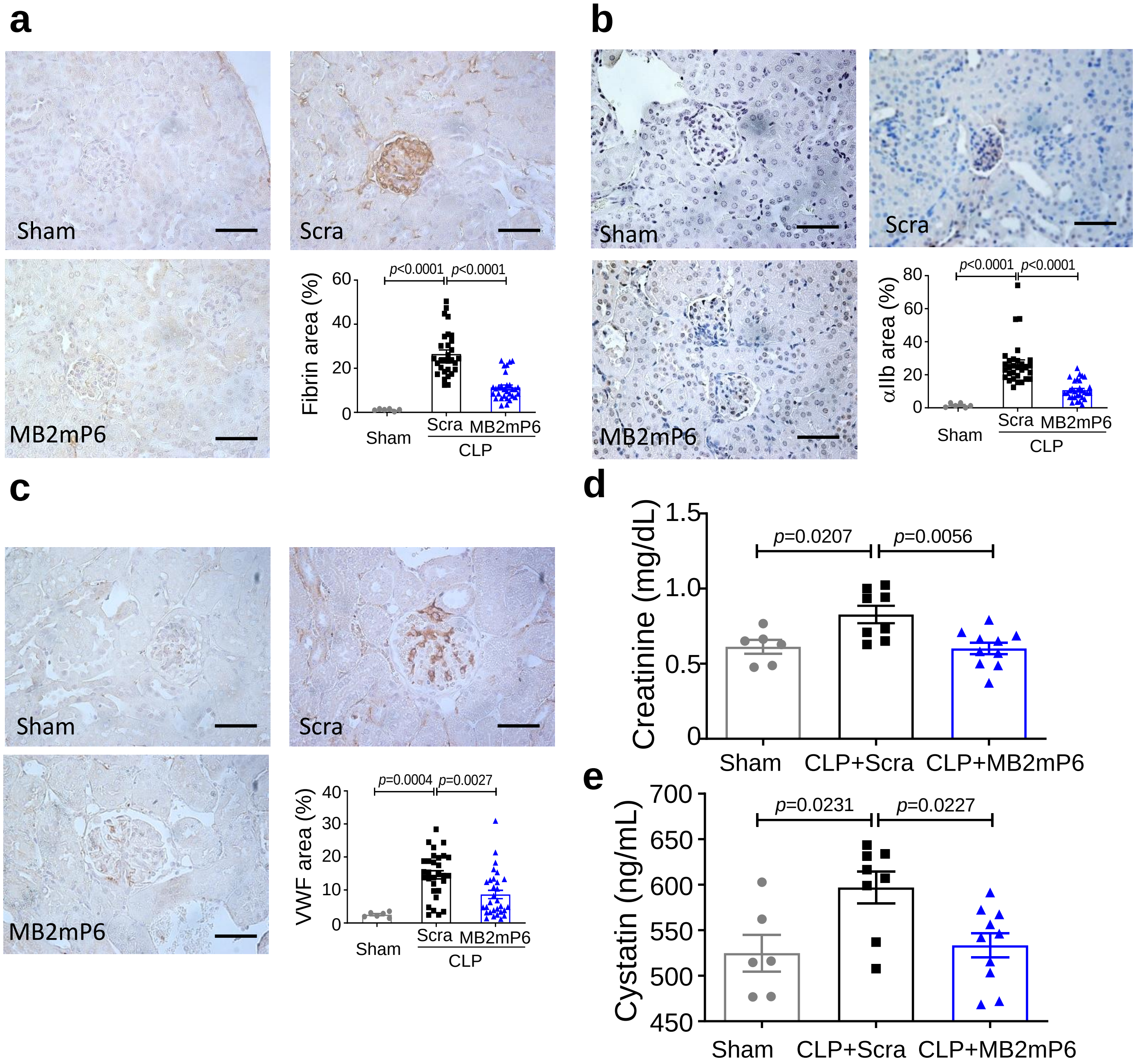

Supplementary Fig. 4. MB2mP6 inhibits mouse kidney glomerular microvascular thrombosis and kidney function impairment in CLP septic mice. **a**, Representative images of immunohistochemistry stain of fibrin in mouse kidney 24 after CLP. Bars indicate the percentage of fibrin positive stained area per glomerulus (30 random glomeruli from 6 mice/group; sham, n=6; Scra, n=30; MB2mP6, n=30, independent glomeruli). Scale bar=100μm. **b**, Representative images of immunohistochemistry stain of platelet α_{IIb} in mouse kidney 24 after CLP. Bars indicate the percentage of α_{IIb} positive stained area per glomerulus (30 random glomeruli from 6 mice/group; sham, n=6; Scra, n=30; MB2mP6, n=30, independent glomeruli). Scale bar=100μm. **c**, Representative images of immunohistochemistry stain of VWF in mouse kidney 24 after CLP. Bars indicate the percentage of VWF positive stained area per glomerulus (30 random glomeruli from 6 mice/group; sham, n=6; Scra, n=30; MB2mP6, n=30, independent glomeruli). Scale bar=100μm. **d**, Serum level of Creatinine in septic mice 24 hours after CLP was inhibited in MB2mP6 treated mice compared to scrambled peptide treated mice (sham, n=6; Scra, n=8; MB2mP6, n=10, independent animals). **e**, Serum level of Cystatin C in septic mice 24 hours after CLP was inhibited in MB2mP6 treated mice compared to scrambled peptide treated mice (sham, n=6; Scra, n=8; MB2mP6, n=10, independent animals). All data are shown as mean ± SEM. Data in **a, b** and **c** were analyzed by two-tailed nonparametric Mann-Whitney test; data in **d** and **e** were analyzed by one-way ANOVA with the post hoc Tukey's multiple comparisons test.

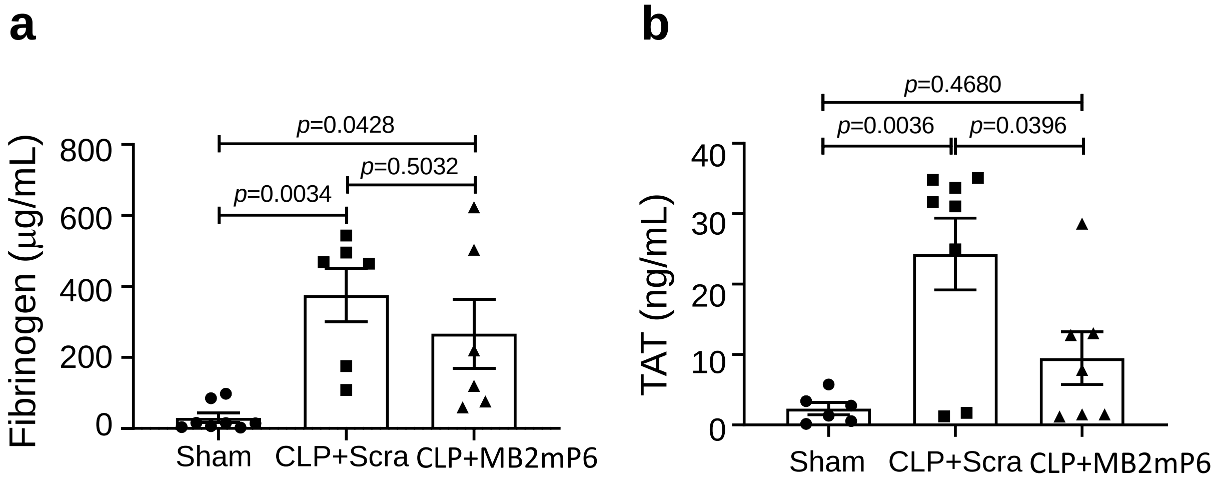

Supplementary Fig. 5. Effects of MB2mP6 on fibrinogen (a) and thrombin-antithrombin complex (TAT) (b) levels in septic mouse blood as measured by ELISA. All data are shown as mean ± SEM (in **a**, sham, n=8; Scra, n=6; MB2mP6, n=6. in **b**, sham, n=6; scra, n=8; MB2mP6, n=7, independent animals) . Data in **a** and **b** were analyzed by one-way ANOVA with the post hoc Tukey's multiple comparisons test.

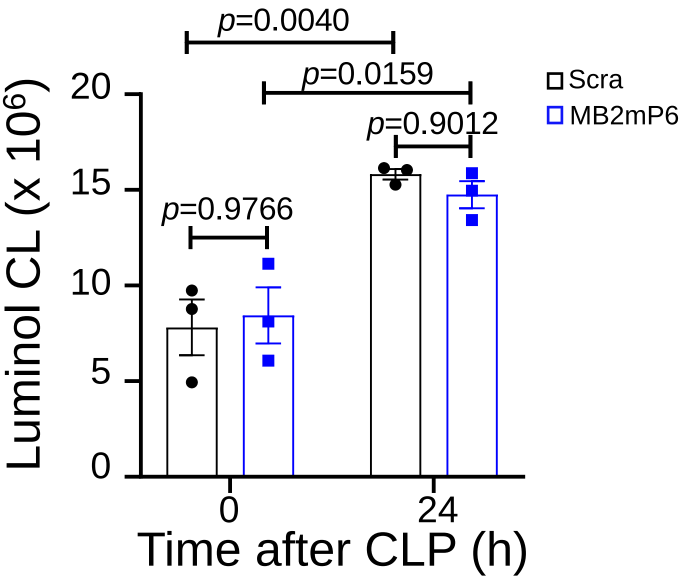

Supplementary Fig. 6. Detection of hemoglobin contents in mouse stools 24 hours after CLP. All data are shown as mean ± SEM (all groups, n=3, independent animals). Data were analyzed by two-way ANOVA with the post hoc Tukey's multiple comparisons test.

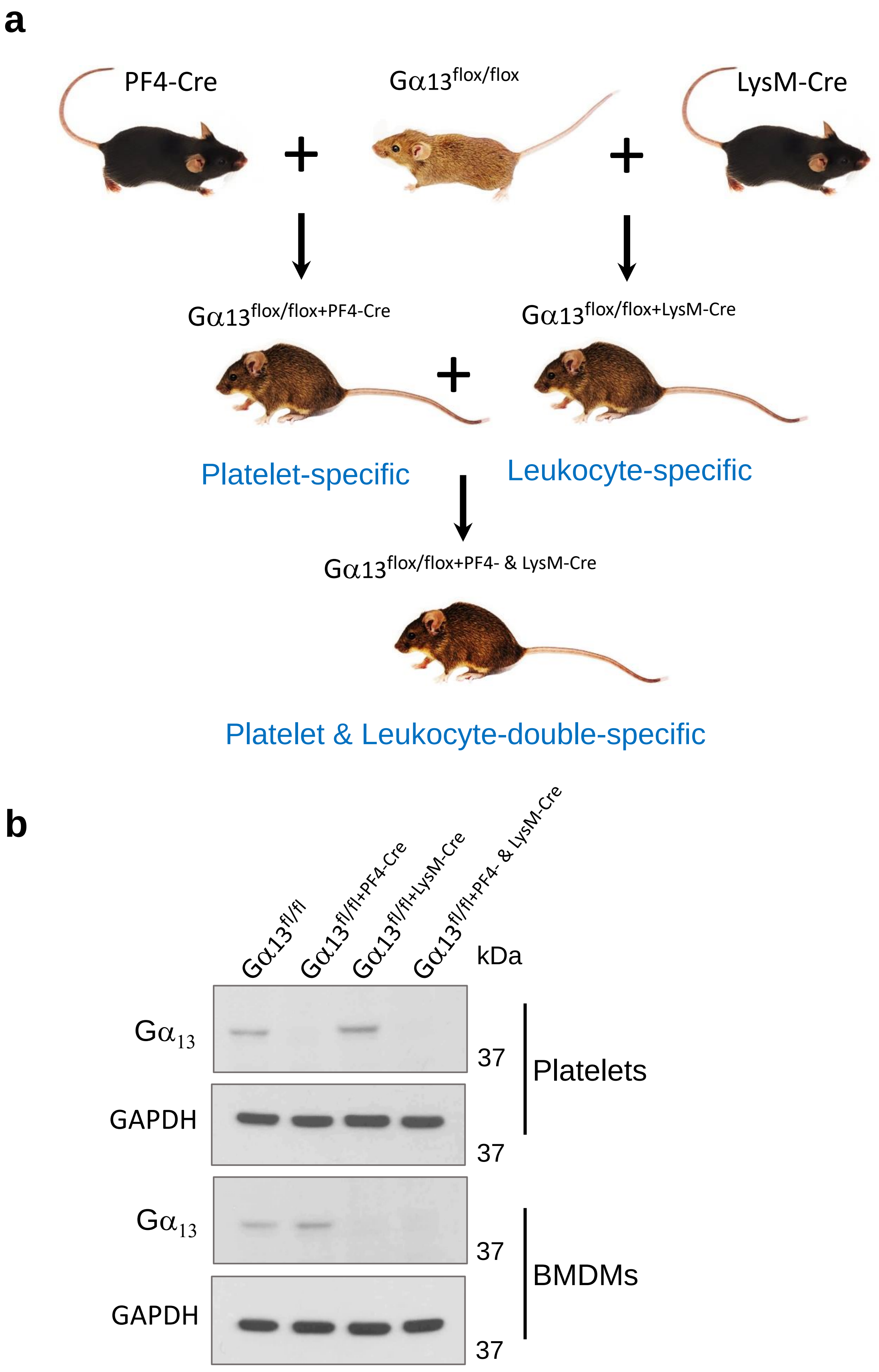

Supplementary Fig. 7. Generation of platelet-specific, leukocyte-specific and platelet & leukocyte-dual-specific Gα_{13} knockout mice. a, schematic breeding strategies for Gα_{13} knockout mice. **b,** Representative western blots for Gα_{13} protein analysis from 4 independent experiments were shown.

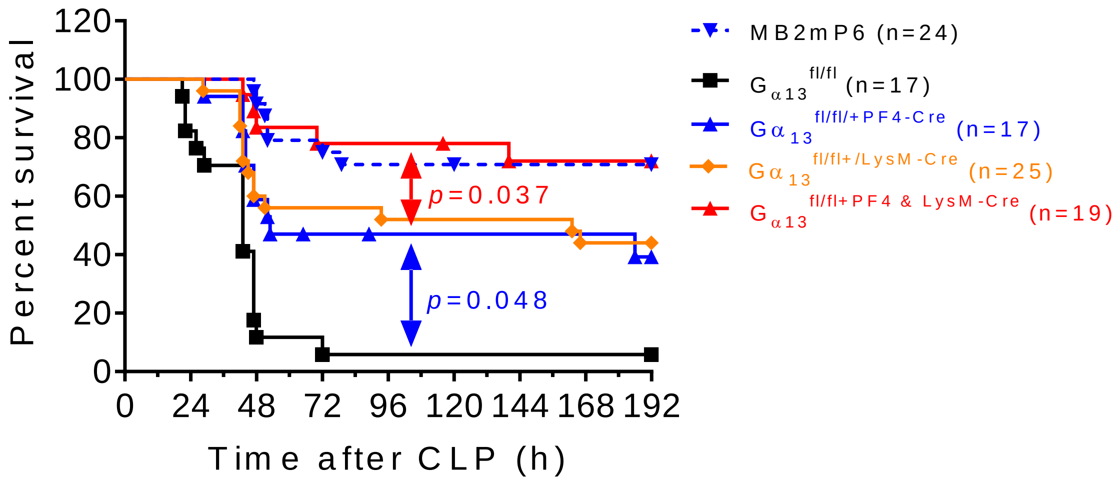

Supplementary Fig. 8. Integrated CLP survival data from the whole study. The survival rate of mice after CLP in MB2mP6 treated C57/BL6 mice (n=24, independent animals), $G\alpha_{13}^{fl/fl}$ (control, n=17, independent animals), $G\alpha_{13}^{fl/fl\text{-}LysMCre}$ (leukocyte-specific $G\alpha_{13}$ knockout mice, n=25, independent animals), $G\alpha_{13}^{fl/fl\text{-}PF4Cre}$ (platelet-specific $G\alpha_{13}$ knockout mice, n=17, independent animals) and $G\alpha_{13}^{fl/fl+PF4\ \&\ LysM\text{-}Cre}$ (leukocyte and platelet dual-specific $G\alpha_{13}$ knockout mice, n=19, independent animals). Log-rank (Mantel-Cox) test using GraphPad Prism software.

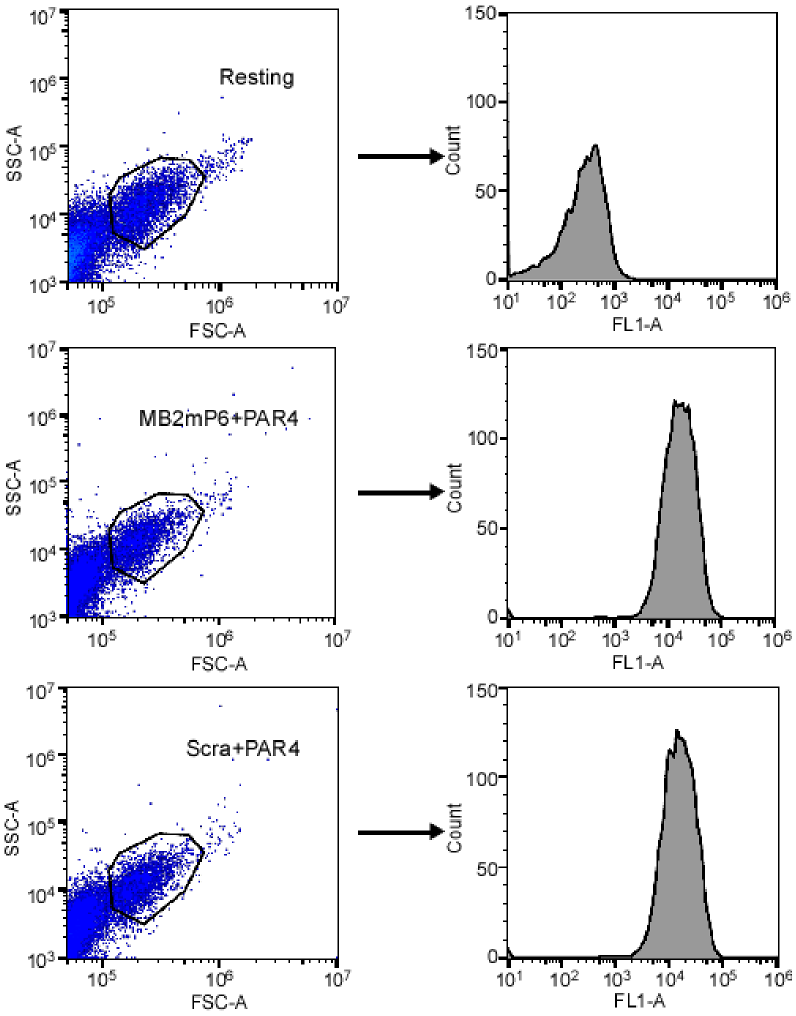

Supplementary Fig. 9. Gating used for flow cytometry analysis. Gating strategy used for Oregon Green-labeled fibrinogen binding to mouse platelets induced by PAR4AP (500 μM). Platelets were pretreated with MB2mP6 or Scrambled peptides HLPN (20 μM) for 10 minutes.

Supplementary Table. 1. QPCR primers used in the study.

Gene name	Primer sequences	
hIL-1b	forward	5'-TTCGACACATGGGATAACGAGG-3'
	reverse	5'-TTTTTGCTGTGAGTCCCGGAG-3'
hIL-6	forward	5'- CCTGAACCTTCCAAAGATGGC-3'
	reverse	5'- TTCACCAGGCAAGTCTCCTCA-3'
hIL-12 P40	forward	5'- GCGGAGCTGCTACACTCTC-3'
	reverse	5'- CCATGACCTCAATGGGCAGAC-3'
hTNFa	forward	5'- GAGGCCAAGCCCTGGTATG-3'
	reverse	5'- CGGGCCGATTGATCTCAGC-3'
hGAPDH	forward	5'- CTGGGCTACACTGAGCACC-3'
	reverse	5'- AAGTGGTCGTTGAGGGCAATG-3'
mIl-1b	forward	5'-CGACAAAATACCTGTGGCCT-3'
	reverse	5'-TTCTTTGGGTATTGCTTGGG-3'
mIl-6	forward	5'-TCCAGTTGCCTTCTTGGGAC-3'
	reverse	5'- GTGTAATTAAGCCTCCGACTTG-3'
mTnfa	forward	5'- CTGAACTTCGGGGTGATCGG-3'
	reverse	5'- GGCTTGTCACTCGAATTTTGAGA-3'
mGapdh	forward	5'- TGCGACTTCAACAGCAACTC-3'
	reverse	5'- CTTGCTCAGTGTCCTTGCTG-3'
