# Supplementary material for: Targeting Gα13-integrin interaction ameliorates systemic inflammation
Source: Nat Commun. 2021 May 27;12:3185. doi: 10.1038/s41467-021-23409-0 (PMC8159967; doi:10.1038/s41467-021-23409-0)
Supplement: Supplementary file 3 — Reporting Summary [file 41467_2021_23409_MOESM3_ESM.pdf]

nature research

Corresponding author(s): Xiaoping Du

Last updated by author(s): Apr 20, 2021

Reporting Summary

Nature Research wishes to improve the reproducibility of the work that we publish. This form provides structure for consistency and transparency in reporting. For further information on Nature Research policies, see our Editorial Policies and the Editorial Policy Checklist.

Statistics

For all statistical analyses, confirm that the following items are present in the figure legend, table legend, main text, or Methods section.

n/a	Confirmed	
☐	☒	The exact sample size (*n*) for each experimental group/condition, given as a discrete number and unit of measurement
☐	☒	A statement on whether measurements were taken from distinct samples or whether the same sample was measured repeatedly
☐	☒	The statistical test(s) used AND whether they are one- or two-sided<br>*Only common tests should be described solely by name; describe more complex techniques in the Methods section.*
☒	☐	A description of all covariates tested
☐	☒	A description of any assumptions or corrections, such as tests of normality and adjustment for multiple comparisons
☐	☒	A full description of the statistical parameters including central tendency (e.g. means) or other basic estimates (e.g. regression coefficient) AND variation (e.g. standard deviation) or associated estimates of uncertainty (e.g. confidence intervals)
☐	☒	For null hypothesis testing, the test statistic (e.g. *F*, *t*, *r*) with confidence intervals, effect sizes, degrees of freedom and *P* value noted<br>*Give P values as exact values whenever suitable.*
☒	☐	For Bayesian analysis, information on the choice of priors and Markov chain Monte Carlo settings
☒	☐	For hierarchical and complex designs, identification of the appropriate level for tests and full reporting of outcomes
☒	☐	Estimates of effect sizes (e.g. Cohen's *d*, Pearson's *r*), indicating how they were calculated

Our web collection on statistics for biologists contains articles on many of the points above.

Software and code

Policy information about availability of computer code

Data collection	Detailed in Methods section. For quantitative PCR, QuanStudio Real-Time PCP Software (version 1.3) from Thermo Fisher Scientific Inc. was used; For FeCl3-induced thrombosis assay, WinDaq HiRes Acquisition (version 3.93) from DATAQ Instruments, Inc. was used; For ELISA and any other colorimetric assays, SoftMAX pro software (version 2.2.1) from Molecular Devices, LLC. was used; For platelet aggregation and secretion assay, Aggro/Link8 for Windows (version 1.2.9) from CHRONO-LOG CORP. was used; For flow cyotmetry analysis, CFlow Plus software (version 1.0.227.4) from BD Biosciences was used.
Data analysis	For IHC image analysis and bacteria colony counting, ImageJ software (version 1.53e) from NIH was used. For data statistic analysis, GarphPad Prism (version 8.3.0) from GraphPad Software and Excel (office 365) from Microsoft, Inc. were used.

For manuscripts utilizing custom algorithms or software that are central to the research but not yet described in published literature, software must be made available to editors and reviewers. We strongly encourage code deposition in a community repository (e.g. GitHub). See the Nature Research guidelines for submitting code & software for further information.

Data

Policy information about availability of data

All manuscripts must include a data availability statement. This statement should provide the following information, where applicable:

- Accession codes, unique identifiers, or web links for publicly available datasets
- A list of figures that have associated raw data
- A description of any restrictions on data availability

The data that support the findings in the current study are available in the Article and its Supplementary Information files. Additional information can be obtained from the corresponding author upon reasonable request.

Field-specific reporting

Please select the one below that is the best fit for your research. If you are not sure, read the appropriate sections before making your selection.

☒ Life sciences ☐ Behavioural & social sciences ☐ Ecological, evolutionary & environmental sciences

For a reference copy of the document with all sections, see nature.com/documents/nr-reporting-summary-flat.pdf

Life sciences study design

All studies must disclose on these points even when the disclosure is negative.

Sample size	Sample sizes were determined according to previous studies published by our group (Ref. 24, 47 and 52) or others. For key experiments, the confidence and sample sizes were evaluated by Power analysis based on the preliminary data using G Power software as previously described (Pang et al, 2020, 12:552, Sci Transl Med). For in vitro or ex vivo studies, all the experiments were independently repeated at least three times to obtain data for statistical analyses. For in vivo studies, mice were assigned into experimental groups on the basis of the genotypes and randomized within given age with equal amount of genders. Per animal protection requirement and when scientifically valid, data collected in identical in vivo experiments are used for multiple purposes to avoid unnecessary mouse sacrifice. Sample sizes and statistical results are shown in figure legends.
Data exclusions	No data were excluded.
Replication	The majority data in the study were repeated at least three times to ensure the reproducibility. Proper statistical analyses were applied to illustrate significance when it's needed. Some Key experiments were repeated by different persons in the group and all the attempts were successful.
Randomization	All animals were grouped on the basis of genotypes within given age with equal amount of genders. All mice in the treatment and control groups were also randomly assigned with similar age and equal amount sexes. For ex vivo experiments, mouse platelets or bone marrow derived macrophages or neutrophils were isolated and pooled from 2-6 mice and treated as one independent experiment.
Blinding	The picture taking and quantification of IHC slides were blinded by covering the label on each slide. The CLP surgeries were performed without knowing individual genotype and the survival data were obtained and grouped by mouse ear tag with corresponding genotype record. The bacteria colony counting was blinded by calculated with different persons. In vitro experiments were not blinded because blinding are not effective in improving objectivity in these experiments and inefficient.

Reporting for specific materials, systems and methods

We require information from authors about some types of materials, experimental systems and methods used in many studies. Here, indicate whether each material, system or method listed is relevant to your study. If you are not sure if a list item applies to your research, read the appropriate section before selecting a response.

Materials & experimental systems

n/a	Involved in the study	
☐	☒	Antibodies
☐	☒	Eukaryotic cell lines
☒	☐	Palaeontology and archaeology
☐	☒	Animals and other organisms
☐	☒	Human research participants
☒	☐	Clinical data
☒	☐	Dual use research of concern

Methods

n/a	Involved in the study	
☒	☐	ChIP-seq
☐	☒	Flow cytometry
☒	☐	MRI-based neuroimaging

Antibodies

Antibodies used	1. Mouse anti-integrin beta2 antibody (1.BB.246, sc-71397) and rat anti-integrin alpha IIb antibody (MWReg30, sc-19963) were purchased from Santa Cruz Biotechnology (Dallas, TX).<br>2. Rabbit anti-integrin beta 2 monoclonal antibody (D4N5Z, #73663) was purchased from Cell Signaling Technology, Inc. (Danvers, MA).<br>3. Mouse anti-integrin beta 3 monoclonal antibody M15 was a gift from Dr. Mark Ginsberg (UCSD, CA).<br>4. Rabbit anti-integrin beta 3 antibody (18309-1-AP) was obtained from Proteintech Group Inc. (Chicago, IL).<br>5. Rabbit anti-Ga13 antibody (GTX32613) was purchased from GeneTex Inc. (Irving, CA).<br>6. Rabbit anti-fibrin/fibrinogen polyclonal antibody (A0080) was obtained from Dako/Agilent (Santa Clara, CA).<br>7. FITC-conjugated rat anti-mouse P-selectin antibody was purchased from BD Pharmingen (RB40.34, 553744).<br>8. Rabbit anti-VWF antibody (AB7356) was purchased from Millipore-Sigma (Kankakee, IL).<br>9. PE/Cyanine7 labeled rat anti-mouse Ly-6G Antibody (clone 1A8, 127618) was purchased from Biolegend (San Diego, CA).

Validation	1. Mouse anti-integrin beta 2 antiboy (1.BB.246) was used to immunoprecipitate beta 2 integrin from human samples. 2 micro gram/500 micro gram lysate was sufficient to IP beta 2 integrin in the present study. Product information show as: https://www.scbt.com/p/integrin-beta2-antibody-1-bb-246.<br><br>2. Rat anti-integrin alpha IIb antibody (MWReg30, sc-19963) was used to stain and identify mouse platelets in IHC staining at dilution of 1:250. The dosage was validated based on previous publication (Ref. 48). Production information show as: https://www.scbt.com/p/integrin-alphaiib-antibody-mwreg30?requestFrom=search.<br><br>3. Rabbit anti-integrin beta 2 monoclonal antibody (D4N5Z, #73663) was used to blot human beta 2 integrin in western blot assay (1:1000). Product information and citations can be found at: https://www.cellsignal.com/products/primary-antibodies/integrin-b2-d4n5z-rabbit-mab/73663<br><br>4. Mouse anti-integrin beta 3 monoclonal antibody M15 as a gift from Dr. Mark Ginsberg's lab was used to immunoprecititate beta 3 integrin from human platelets. The dosage of M15 in co-IP assay (2 micro gram / 500 micro gram cell lysate) was validated in our previous publication (Ref. 25)<br><br>5. Rabbit anti-integrin beta 3 antibody (18309-1-AP) was used to blot human beta 3 integrin in western blot assay (1:1000). Product information and citations can be found at: https://www.ptglab.com/products/ITGB3-Antibody-18309-1-AP.htm.<br><br>6. Rabbit anti-Ga13 antibody (GTX32613) was used to blot Ga13 in western blot assay (1:1000). The dilution time was validated in our previous publication (Ref. 25). The product information and citations can be found at: https://www.ptglab.com/products/ITGB3-Antibody-18309-1-AP.htm.<br><br>7. Rabbit anti-fibrin/fibrinogen polyclonal antibody (A0080) was used to identify fibrin deposition in IHC staining (1:2,000). The usage of A0080 was validated by several labs including Akassoglou's lab (https://akassogloulab.org/wp-content/uploads/2019/04/Fibrinogen-Immunohistochemistry-Mouse-PROTOCOL.pdf). The product information can be found at: https://www.agilent.com/search/?filter=AND(SpSearch:TechSupport,p_product_id:PRDT_76975_en_GLOBAL).<br><br>8. FITC-conjugated rat anti-mouse P-selectin antibody (553744) was used to label and detect platelet surface P-selectin expression. The product information, applications and citations can be found at: https://www.bdbiosciences.com/eu/applications/research/t-cell-immunology/regulatory-t-cells/surface-markers/mouse/fitc-rat-anti-mouse-cd62p-rb4034/p/553744.<br><br>9. Rabbit anti-VWF antibody (AB7356) was used to stain VWF in IHC staining (1:50). The validation of the antibody and other product information can be found at: https://www.emdmillipore.com/US/en/product/Anti-von-Willebrand-Factor-Antibody,MM_NF-AB7356.<br><br>10. PE/Cyanine7 labeled rat anti-mouse Ly-6G Antibody (clone 1A8, 127618) was used to label mouse neutrophils as indicated at: https://www.biolegend.com/en-us/products/pe-cyanine7-anti-mouse-ly-6g-antibody-6139?GroupID=GROUP20.

Eukaryotic cell lines

Policy information about cell lines

Cell line source(s)	Human monocytic leukemia cell line THP-1 was purchased from ATCC (TIB-202).
Authentication	The cell line was not authenticated.
Mycoplasma contamination	THP-1 cells were negative of mycoplasma contamination by Mycoplasma PCR test.
Commonly misidentified lines (See ICLAC register)	THP-1 cells are not misidentified cell lines.

Animals and other organisms

Policy information about studies involving animals; ARRIVE guidelines recommended for reporting animal research

Laboratory animals	Mouse colony C57BL/6J (stock # 000664), LysMcre (stock # 004781) and PF4cre (stock # 008535) were purchased from Jackson Laboratory. The Ga13 flox/flox mice were a gift from Dr. Stefan Offermanns' lab (Max Planck Institute for Heart and Lung Research, Bad Nauheim, Germany). All the mice of 8- to 16-weeks-old with an equal sex ratio were used in the studies and housed and bred in Biologic Resources Laboratory at University of Illinois at Chicago under 12 hours light dark cycles, control temperature (~23 degree) and 40-50% humidity with free access to food and water.
Wild animals	This study did not involve the use of wild animals.
Field-collected samples	This study did not involve the use of field-collected samples.
Ethics oversight	Animal usage and protocol were approved by the Institutional Animal Care Committee, University of Illinois at Chicago. A randomized approach of choosing mice was used throughout the study, using all mice with the correct genotype without bias.

Note that full information on the approval of the study protocol must also be provided in the manuscript.

Human research participants

Policy information about studies involving human research participants

Population characteristics	Adults of either sex with any ethnic category who are in good health.
Recruitment	The healthy donors were randomly recruited without any self-selection by the performer. Blood drawings were performed by a certified phlebotomist.
Ethics oversight	Human Subjects Research Protocol was approved by IRB of Office for the Protection of Research Subjects at University of Illinois at Chicago.

Note that full information on the approval of the study protocol must also be provided in the manuscript.

Flow Cytometry

Plots

Confirm that:

☒ The axis labels state the marker and fluorochrome used (e.g. CD4-FITC).

☒ The axis scales are clearly visible. Include numbers along axes only for bottom left plot of group (a 'group' is an analysis of identical markers).

☒ All plots are contour plots with outliers or pseudocolor plots.

☒ A numerical value for number of cells or percentage (with statistics) is provided.

Methodology

Sample preparation	Described in detailed in Methods part.
Instrument	Accuri C6 flowcytometry.
Software	CFlow Plus software (version 1.0.227.4) from BD Biosciences for collection and Flow Jo (Version 7) for analysis.
Cell population abundance	90~95% of live human and mouse platelets were validated by anti- CD41 or CD42b staining. ~80% of isolated mouse neutrophils were validated by PE-Ly6G labeling.
Gating strategy	Platelets gating strategy was routinely used in our lab as published in various publications such as (Zhang G, et al, J Immunol. 2009, 182(12):7997-8004) and (Li Z, et al, J Biol Chem. 2004, 279(41):42469-75).

☒ Tick this box to confirm that a figure exemplifying the gating strategy is provided in the Supplementary Information.
